# Clinical and dermoscopic analysis of 32 cases of acquired facial hyperpigmented macules

**DOI:** 10.3389/fmed.2026.1855328

**Published:** 2026-06-16

**Authors:** Xiaoyi Chen, Yao Wu, Jing Xiao, Liansheng Zhong

**Affiliations:** 1 Department of Dermatology, Xiamen Children’s Hospital (Children’s Hospital of Fudan University at Xiamen), Xiamen, China; 2 Department of Dermatology, Children’s Hospital of Fudan University & National Children Medical Center, Shanghai, China; 3 Fujian Key Laboratory of Neonatal Disease, Xiamen Children’s Hospital (Children’s Hospital of Fudan University at Xiamen), Xiamen, China

**Keywords:** acquired facial hyperpigmented macules, dermoscopy, foreheads, temples, ultraviolet

## Abstract

**Background:**

Acquired facial hyperpigmented macules (AFHM), which appear as non-segmental pigmented macules without a history of obvious localized inflammation, usually occur on the foreheads and temples of young children. Currently, the etiology and pathogenesis of AFHM remain unclear.

**Objective:**

To investigate the clinical and dermoscopic features of AFHM.

**Methods:**

A retrospective study was conducted on the clinical and dermoscopic features of 32 children with AFHM.

**Results:**

The lesions of AFHM are light brown macules ranging in size from millet to peanut, without any symptoms, mainly distributed on the foreheads and temples. Light brown pseudoreticular pigment and linear/branching vessels are the two major dermoscopic features of AFHM. During the follow-up period, which ranged from 31 months to 67 months, the lesions of all patients improved, and 27 of the 28 followed patients (96.4%) showed complete disappearance of the lesions.

**Conclusion:**

Acquired facial hyperpigmented macules is a self-limited disease characterized by sudden appearance of irregularly shaped pigmented macules on the foreheads and temples in young children. We propose a hypothesis that ultraviolet radiation may play an important role in the etiology and pathogenesis of AFHM.

## Introduction

Acquired facial hyperpigmented macules (AFHM), which appear as non-segmental pigmented macules without a history of obvious localized inflammation, usually occur on the foreheads and temples of young children. Currently, the etiology and pathogenesis of AFHM remain unclear. In this report, we analyzed the clinical and dermoscopic features of 32 patients with AFHM and proposed a hypothesis that ultraviolet radiation may play an important role in the etiology and pathogenesis of AFHM.

## Methods

We performed a retrospective study of the clinical and dermoscopic features of 32 patients with AFHM who visited the dermatology outpatient department at Xiamen Children’s hospital (Children’s Hospital of Fudan University at Xiamen) from December 2019 to June 2023. The diagnosis of AFHM was mainly based on the following points: ① Onset in infancy; ② Lesions located on the forehead and temporal regions; ③ The skin lesions are generally multiple, with a diameter mostly less than 1 cm; ④ Dermoscopy usually shows pseudoreticular pigmentation, with or without linear vessels. Patients with pigmentation present at birth, or those who could not rule out other pigmented disorders such as melasma, freckles, pigmented urticaria, tinea versicolor, café-au-lait spots, post-inflammatory hyperpigmentation and Riehl melanosis, were excluded. We selected patients diagnosed with AFHM from our daily medical records. Two dermatologists reviewed the cases and confirmed the diagnosis. General information regarding gender, age of onset, season of onset, and disease course was collected. The characteristics of the lesions, laboratory test results, and prognosis were also analyzed. Although the patients were followed up by telephone, we ensured that the data were accurate because the questions were quite simple, and we repeatedly asked, using different phrasings, to confirm whether skin lesions had completely disappeared or improved. All study protocols have been approved by Ethics Committee of Xiamen Children’s Hospital (Approval no. [2022]04) and all parents of the patients signed informed consent before data collection.

## Statistical analysis

We used SPSS 21.0 software to analyze the data. Quantitative data are described using the mean and standard deviation, qualitative data are described by percentage.

## Results

### General information

Age at presentation ranged from 5 months to 34 months (12.5 ± 5.7 months). Seventeen cases (53.1%) were male, and 15 cases (46.9%) were female. Disease duration ranged from 1 month to 19 months (5.4 ± 4.6 months). The age of onset ranged from 1 month to 15 months (7.0 ± 3.9 months), with 30 cases (93.8%) occurring in infancy (<1 year). The seasons of onset were as follows: spring (2 cases, 6.3%),summer (7 cases, 21.9%), autumn (12 cases, 37.5%), and winter (11 cases, 34.3%). The patients’ general information is also summarized in Table 1.

**TABLE 1:** General information of 32 patients with AFHM.

General information	Values
Age at presentation	5–34 (12.5 ± 5.7) months
Female	15
Age of onset	1–15 (7.0 ± 3.9) months
Disease duration	1–19 (5.4 ± 4.6) months
Seasons of onset	
Spring	2, 6.3%
Summer	7, 21.9%
Autumn	12, 37.5%
Winter	11, 34.3%

### Clinical characteristics

**FIGURE 1:**
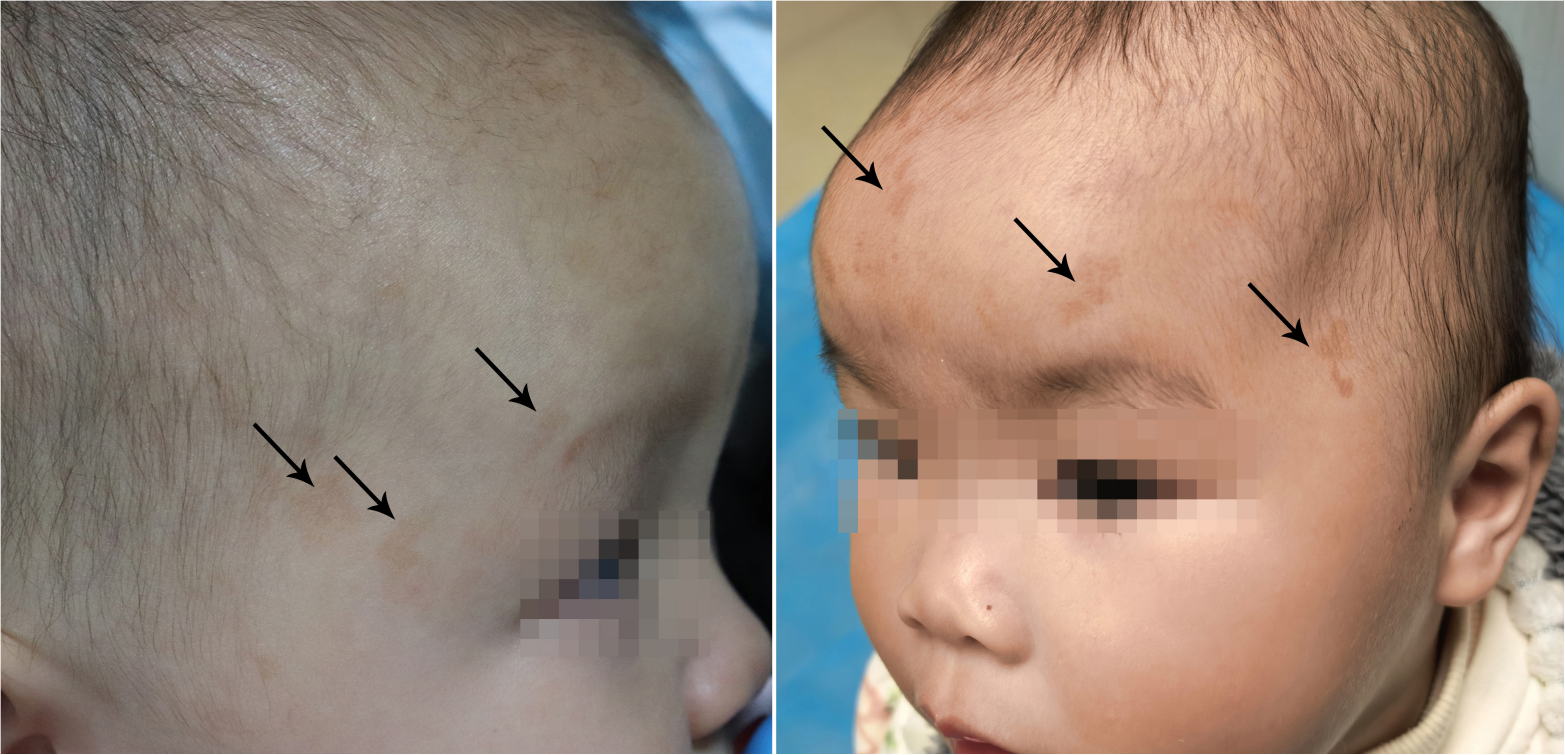
Lesions of AFHM. Multiple randomly distributed irregular hyperpigmented macules on the forehead and temple of young children (black arrows).

The lesions of AFHM are light brown macules without any symptoms, irregular in shape, and range in size from millet to peanut (Figure 1). There were no papules, wheals, desquamation, or other skin lesions. The forehead (68.8%, *n* = 22) was the most common site in these patients, followed by the temple (9.4%, *n* = 3), the zygoma (6.2%, *n* = 2) and the cheek (3.0%, *n* = 1), both the forehead and temple were involved in 3 cases (9.4%) and both the forehead and cheek in 1 case (3.0%). The lesions became red after crying in 12 cases and after exposure to sunlight in 14 cases. The number of lesions varied from 3 to 12, with either bilateral or unilateral distribution. Darier’s sign was negative in all patients. Fungal microscopy was performed in 3 patients in whom tinea versicolor could not be ruled out based on clinical manifestations, and all showed negative results. We advised sun protection for all patients, and the main interventions recommended were physical photoprotection and reducing the duration of sun exposure. The clinical characteristics of the 32 patients were also summarized in Table 2.

**TABLE 2:** Clinical and dermoscopic characteristics of 32 patients with AFHM.

Clinical and dermoscopic characteristics	Values
Lesion distribution	
Unilateral	12
Bilateral	20
Lesion location	
Forehead	26
Temple	6
Zygoma	2
Cheek	2
Number of lesions	3–12 (5.9 ± 2.2)
Lesions become red after crying	12
Lesions become red after sunlight	14
Sun protection history	
Yes	27
No	5
Family history	
Yes	3
No	29
Follow-up duration (***N*** = 28)	31–67 (45.5 ± 10.2) months
Lesions duration (***N*** = 28)	5–70 (19.5 ± 15.5) months
Final outcome (N = 28)	
Complete disappearance	27
Improve	1
Dermoscopic findings (N = 22)	
Pseudoreticular pigmentation	22
Linear/branching vessels	20

### Dermoscopy findings

**FIGURE 2:**
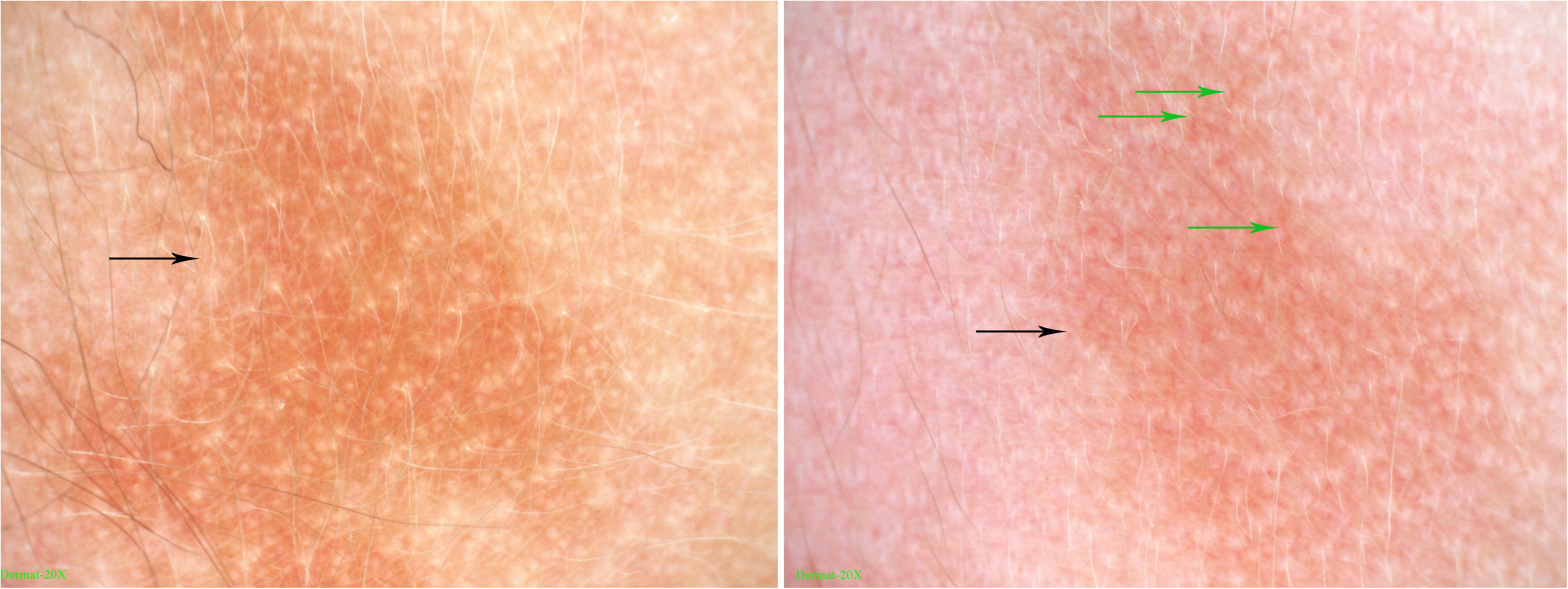
Dermoscopy of the lesions of AFHM. Light brown pseudoreticular pigmentation (black arrows) and linear vessels (green arrows) under dermoscopy.

Twenty-two (68.8%) patients underwent dermoscopy examination using a Dermoscopy Image Diagnose Workstation (DERMOSCOPY-II; Beijing Dermat Speedy Recovery T&D Co., Ltd., Peking, China) with a magnification of 50×, both polarized and non-polarized modes were used, and photographs of the area of interest were obtained. dermoscopic images were reviewed by two dermatologists. All of them showed light brown pseudoreticular pigmentation, and 20 (90.9%) patients showed linear/branching vessels (Figure 2). Since most patients with AFHM can be diagnosed on clinical manifestations alone, some patients did not undergo dermoscopy examination. The dermoscopic characteristics of the 32 patients are also summarized in Table 2.

### Follow up

A total of 28 patients (87.5%) completed telephone follow-up, with the follow-up duration ranging from 31 to 67 months. The lesions of all patients improved, and 27 of the 28 followed patients (96.4%) showed complete disappearance of the lesions, which usually occurred within 2 years. One patient’s lesions disappeared after 3 months of sun protection, but recurred after 2 months of neglecting sun protection, then disappeared again after 2 months of sun protection. Another patient’s lesions persisted for 31 months without sun protection, but after 2 months of sun protection, the lesions completely disappeared.

## Discussion

Hyperpigmentation is one of the most common dermatological complaints among patients with skin of color, especially facial hyperpigmentation, the common causes of facial hyperpigmentation are melasma, post-inflammatory hyperpigmentation, lentigines, ephelides, periorbital melanosis and so on (1). Hernández-Martín et al. (2) were the first to characterize AFHM in 2014, reporting 25 young children with sudden-onset hyperpigmented macules on the foreheads and temples in the absence of preceding inflammation. The clinical features of the 32 patients in this study were consistent with those reported by Hernández-Martín et al. 19 patients in the study of Hernández-Martín et al. were followed up for 3 months to 4.5 years, among them, 14 cases showed no change in lesions, and 5 cases showed partial regression. In this study, a total of 28 patients (87.5%) completed telephone follow-up, with the follow-up duration ranging from 31 months to 67 months. The lesions of all 28 followed patients improved, and 27 of them (96.4%) showed complete disappearance of the lesions, which usually occurred within 2 years. Therefore, we believe that AFHM is a self-limited disease.

To date, the etiology and pathogenesis of AFHM remain unclear. The three leading causes of skin pigmentation are sun exposure, genetics, and medications. Sun exposure is the primary etiological factor for hyperpigmentation, as it potently upregulates melanin biosynthesis pathways (3). Infant skin has low melanin, immature skin barrier function, and poor UV protection, so infants are more vulnerable to UV-induced melanin synthesis. Ko et al. (4) recommended photoprotection for all patients with hyperpigmented disorders. In 2016, Giacaman et al. (5) reported 3 cases of AFHM, among them 2 cases were sisters. In this study three pairs of patients come from the same families, including a pair of twins. These findings suggest that AFHM may have common environmental triggering factors and genetic susceptibility. In this study, the lesions of 14 cases (43.8%) became red after exposure to sunlight. One patient’s lesions disappeared after 3 months of sun protection but recurred after 2 months of neglecting sun protection, then disappeared again after 2 months of sun protection. Another patient’s lesions persisted for 31 months without sun protection, but after strict sun protection for 2 months, the lesions completely disappeared. Therefore, based on the observations of this study, we propose the hypothesis that ultraviolet radiation may play an important role in the etiology and pathogenesis of AFHM, and that sun protection may be a reasonable conservative management strategy. However, further studies are required to confirm this hypothesis.

Dermatoscopy plays an important role in the diagnosis of pigmented skin diseases, such as solar lentigines, melasma, postinflammatory hyperpigmentation, tinea versicolor and Riehl melanosis (6). In 2023, Lai et al. (7) summarized two major dermoscopic features of AFHM: Light brown pseudoreticular pigment and linear/branching vessels. In this study, Dermoscopic evaluation was performed in 22 patients (68.8% of the cohort), all of them showed light brown pseudoreticular pigmentation, and 20 (90.9%) patients showed linear/branching vessels.

AFHM needs to be differentiated from many other skin diseases, such as melasma, freckles, pigmented urticaria, tinea versicolor, café-au-lait spots, post-inflammatory hyperpigmentation and Riehl melanosis. Melasma can also demonstrate a pseudoreticular pigment network along with increased vascularity on dermoscopy, particularly in mixed or dermal variants. But melasma often presents with a more diffuse pigmentation pattern and may include telangiectatic components, and it is more common in young and middle-aged women, commonly affecting the forehead, cheeks, nose, upper lip, chin and mandibular area. Freckles are typically hyperpigmented macules that can appear transiently and darken with sun exposure. However, freckles most commonly occur on the cheeks and nasal bridge, with the lesions typically being smaller (1–3 mm), and dermoscopically may show a reticular or globular pattern. Pigmented urticaria is usually associated with a positive Darier sign; tinea versicolor is usually positive on fungal microscopy examination; Café-au-lait spots show light brown pigmentation with clear boundaries and no apparent vascular structures on dermatoscopy examination; post-inflammatory hyperpigmentation usually occurs after local inflammation, trauma, desquamation or a history of medication use; Riehl melanosis primarily affects individuals with darker skin, especially elderly women, and is characterized by the appearance of brown to gray, reticulate or diffuse pigmented patches on the face, neck, and upper chest.

Limitations: The follow-up data were obtained from the parental reports, without objective confirmation, the reliability is uncertain, however, we ensured that the data were accurate because the follow-up questions were quite simple, and we repeatedly asked, using different phrasings, to confirm whether skin lesions had completely disappeared or improved. Histopathological examination can help distinguish AFHM from other pigmented disorders on the face of children. However, due to the young age of the patients, their parents refused invasive procedures. Therefore, no histopathological examination was performed on the 32 patients in this study.

## Conclusion

The result of this study showed that AFHM is a self-limited disease characterized by the abrupt onset of irregularly shaped hyperpigmented macules localized to the forehead and temporal regions in young children, with no preceding inflammatory manifestations such as erythema, edema, or scaling. We propose the hypothesis that ultraviolet radiation may play an important role in the etiology and pathogenesis of AFHM, and that sun protection may be a reasonable conservative management strategy. However, further studies are required to confirm this hypothesis.

## Data Availability

The original contributions presented in this study are included in this article/supplementary material, further inquiries can be directed to the corresponding author.
